# Ripple-assisted adsorption of noble gases on graphene at room temperature

**DOI:** 10.1093/nsr/nwaf506

**Published:** 2025-11-14

**Authors:** Weilin Liu, Xianlei Huang, Li-Guo Dou, Qianglong Fang, Ang Li, Guowen Yuan, Yongjie Xu, Zhenjia Zhou, Jun Li, Yu Jiang, Zichong Huang, Zihao Fu, Peng-Xiang Hou, Chang Liu, Jinlan Wang, Wu Zhou, Ming-Gang Ju, Shao-Chun Li, Hui-Ming Cheng, Libo Gao

**Affiliations:** National Laboratory of Solid State Microstructures, Jiangsu Key Laboratory for Nanotechnology, Jiangsu Physical Science Research Center, School of Physics, Nanjing University, Nanjing 210093, China; National Laboratory of Solid State Microstructures, Jiangsu Key Laboratory for Nanotechnology, Jiangsu Physical Science Research Center, School of Physics, Nanjing University, Nanjing 210093, China; National Laboratory of Solid State Microstructures, Jiangsu Key Laboratory for Nanotechnology, Jiangsu Physical Science Research Center, School of Physics, Nanjing University, Nanjing 210093, China; Key Laboratory of Quantum Materials and Devices of Ministry of Education, School of Physics, Southeast University, Nanjing 210096, China; School of Physical Sciences, University of Chinese Academy of Sciences, Beijing 101408, China; National Laboratory of Solid State Microstructures, Jiangsu Key Laboratory for Nanotechnology, Jiangsu Physical Science Research Center, School of Physics, Nanjing University, Nanjing 210093, China; National Laboratory of Solid State Microstructures, Jiangsu Key Laboratory for Nanotechnology, Jiangsu Physical Science Research Center, School of Physics, Nanjing University, Nanjing 210093, China; National Laboratory of Solid State Microstructures, Jiangsu Key Laboratory for Nanotechnology, Jiangsu Physical Science Research Center, School of Physics, Nanjing University, Nanjing 210093, China; National Laboratory of Solid State Microstructures, Jiangsu Key Laboratory for Nanotechnology, Jiangsu Physical Science Research Center, School of Physics, Nanjing University, Nanjing 210093, China; National Laboratory of Solid State Microstructures, Jiangsu Key Laboratory for Nanotechnology, Jiangsu Physical Science Research Center, School of Physics, Nanjing University, Nanjing 210093, China; National Laboratory of Solid State Microstructures, Jiangsu Key Laboratory for Nanotechnology, Jiangsu Physical Science Research Center, School of Physics, Nanjing University, Nanjing 210093, China; National Laboratory of Solid State Microstructures, Jiangsu Key Laboratory for Nanotechnology, Jiangsu Physical Science Research Center, School of Physics, Nanjing University, Nanjing 210093, China; Shenyang National Laboratory for Materials Sciences, Institute of Metal Research, Chinese Academy of Sciences, Shenyang 110016, China; Shenyang National Laboratory for Materials Sciences, Institute of Metal Research, Chinese Academy of Sciences, Shenyang 110016, China; Key Laboratory of Quantum Materials and Devices of Ministry of Education, School of Physics, Southeast University, Nanjing 210096, China; Suzhou Laboratory, Suzhou 215000, China; School of Physical Sciences, University of Chinese Academy of Sciences, Beijing 101408, China; Key Laboratory of Quantum Materials and Devices of Ministry of Education, School of Physics, Southeast University, Nanjing 210096, China; National Laboratory of Solid State Microstructures, Jiangsu Key Laboratory for Nanotechnology, Jiangsu Physical Science Research Center, School of Physics, Nanjing University, Nanjing 210093, China; Hefei National Laboratory, Hefei 230026, China; Shenyang National Laboratory for Materials Sciences, Institute of Metal Research, Chinese Academy of Sciences, Shenyang 110016, China; Institute of Technology for Carbon Neutrality, Shenzhen Institute of Advanced Technology, Chinese Academy of Sciences, Shenzhen 518055, China; National Laboratory of Solid State Microstructures, Jiangsu Key Laboratory for Nanotechnology, Jiangsu Physical Science Research Center, School of Physics, Nanjing University, Nanjing 210093, China; State Key Laboratory of Chemo/Biosensing and Chemometrics, Key Laboratory for Micro-Nano Physics and Technology of Hunan Province, College of Materials Science and Engineering, Hunan University, Changsha 410082, China

**Keywords:** noble gases, stable adsorption, ripple, graphene, room temperature

## Abstract

Controllable gas adsorption is critical for both scientific and industrial fields, and high-capacity adsorption of gases on solid surfaces provides significant promise due to its high safety and low energy consumption. However, the adsorption of nonpolar gases, particularly noble gases, poses a considerable challenge under atmospheric pressure and room temperature (RT). Here, we theoretically simulate and experimentally realize the stable adsorption of noble gases like xenon (Xe), krypton (Kr), argon (Ar), and helium (He) on highly rippled graphene at RT. The elemental characteristics of adsorbed Xe are confirmed by electron energy loss spectroscopy and X-ray photoelectron spectroscopy. The adsorbed gas atoms are crystallized with periodic arrangements. These adsorbed noble gases on graphene exhibit high stability at RT and can be completely desorbed at ∼350°C without damaging the intrinsic lattice of the graphene. The structural and physical properties of graphene are significantly influenced by the adsorbed gas, and they fully recover after desorption. Additionally, this controllable adsorption could be generalized to other layered adsorbents such as NbSe_2_, MoS_2_, and carbon nanotubes. We anticipate that this ripple-assisted adsorption will not only re-define the theoretical framework of gas adsorption, but also accelerate advancements in gas storage and separation technologies, as well as enhance the applications in catalysis, surface modification, and other related fields.

## Teaser

Room temperature adsorption and crystallization of noble gases on rippled graphene enables a new path toward revolutionizing gas catalysis, stroage and separation.

## INTRODUCTION

Adsorption theory has evolved over a century from Freundlich’s empirical formula to Langmuir’s isothermal model and the Brunauer-Emmett-Teller (BET) theory [1–4], maturing into a well-established framework where the adsorption of gas molecules on solid surfaces is categorized into chemisorption and physisorption. Chemisorption involves high interaction energy, and the resultant chemical bonds usually lead to the degradation of adsorbents [5–8]. In contrast, physisorption is considered to be reversible with low regeneration energy consumption, the structural and chemical properties of the adsorbent are basically unchanged, and most gas molecules interact with the solid surface through weak van der Waals (vdW) interactions. Solid-gas interface adsorption underlies numerous significant phenomena and applications, including gas storage and separation [9–12], surface catalysis [6,13,14], modification of the adsorbents, and vapour growth of crystals [15–17].

Among all gas molecules, nonpolar gases, especially noble gases such as xenon (Xe), krypton (Kr), argon (Ar), and helium (He), are challenging for stable adsorption on solid surfaces under normal conditions, i.e. atmospheric pressure (AP) and room temperature (RT). This difficulty arises from their fully occupied orbitals, which result in saturated electronic charges and weak interactions with solid surfaces. Some experimental efforts have been made to improve the adsorption ability of noble gases, mainly including lowering temperature and utilizing materials with a high specific surface area as adsorbents [9,10,16–23]. For instance, He atoms have been successfully adsorbed onto graphite at a temperature of 120 mK [21], and Ar, Kr, and Xe atoms have also been adsorbed on a surface-confined metal-organic framework (MOF) at temperatures lower than 8 K [24]. In these cases, the low temperature adsorption of noble gases is classified as physisorption. However, these adsorbed noble gas atoms are restricted to cryogenic temperatures, and desorption occurs spontaneously once the temperature exceeds the critical thresholds. To date, stable adsorption of noble gas atoms under normal conditions has not been experimentally achieved, and theoretical guidance remains inadequate.

## RESULTS AND DISCUSSION

### Realization of noble gas atoms adsorbing on rippled graphene

**Figure 1.**
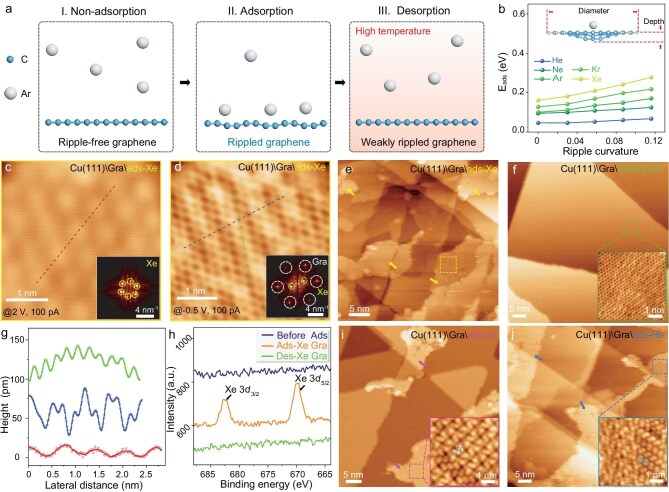
Noble gas atoms adsorbed on rippled graphene. (a) Schematic of noble gas atoms adsorbing on rippled graphene. Left (I. non-adsorption) illustrates no Ar atoms adsorbed on absolutely flat graphene under normal conditions; middle (II. adsorption) illustrates the stable adsorption of Ar atoms on the highly rippled graphene; right (III. desorption) illustrates the desorbing of Ar from graphene at high temperature. Here Ar represents the noble gas atoms. (b) Theoretically calculated *E*_ads_ of graphene with different ripple curvatures adsorbing one noble gas atom. Inset illustrates the side view of rippled graphene (96 carbon atoms) adsorbing one Ar atom. The ripple curvature is the ratio of height to lateral diameter. (c, d) Typical STM topographic images of ads-Xe on Cu(111)\Gra measured under different bias voltages. They are collected from the dashed box labelled in (e). (e) Large-scale STM topographic image of partially ads-Xe on Cu(111)\Gra. The irregular domains labelled by arrows are formed by ads-Xe. Insets are their corresponding FFT patterns. (f) Typical STM image of Cu(111)\Gra after full desorption via thermal annealing, revealing a clean surface free of adsorbed gases. The inset shows a zoom-in image of the graphene lattice, highlighting its defect-free structure. (g) Height profiles of ads-Xe graphene collected from the dashed lines in (c) and (d). The lateral distance between the neighbouring Xe atoms is ∼6.8 Å. (h) Fine XPS spectra of as-transferred graphene, ads-Xe, and des-Xe graphene. The characteristic Xe peaks of 3*d*_3/2_ and 3*d*_5/2_ core levels emerge. (i, j) Typical STM topographic images of partially ads-Ar (i) and ads-He (j) on Cu(111)\Gra. The irregular domains are formed by the adsorbed Ar and He atoms, and the insets are the zoom-in images of the Ar and He domains.

Figure 1a illustrates the schematic of adsorbing noble gas atoms on graphene, using Ar as an example. Due to the weak, noble Ar atoms typically cannot adsorb on graphene which has an absolutely flat surface under normal conditions (Scene I. Non-adsorption). However, graphene inherently exhibits intrinsic ripples [25,26], a consequence of thermodynamic stability in two-dimensional (2D) structures, which induce slight out-of-plane deformations in the *sp*^2^ planar lattice, with typical amplitudes of 0.7 Å and corrugation diameter of ∼80 Å [25]. Recent studies have suggested that these intrinsic ripples can reduce the catalytic barrier of H_2_ molecules and dissociate them into atoms [27–29]. Considering that gas adsorption is a prerequisite for surface catalysis, we suppose the enhanced ripples can also act to reduce the adsorption energy. Based on these insights, we demonstrate the stable adsorption of noble gas atoms on highly rippled graphene (Scene II. Adsorption), where no covalent bonds are formed between carbon and Ar atoms. Besides, due to the strong ripple perturbation at elevating temperature, the adsorbed Ar atoms will be readily desorbed from the rippled graphene (Scene III. Desorption).

Next, we theoretically calculated the total adsorption energies (*E*_ads_) of graphene with varying ripple curvatures and one adsorbed noble gas atom. The negative *E*_ads_ values confirm the exothermic adsorption, with more negative values indicating greater energy release and higher stability. For clarity, the absolute values of *E*_ads_ are employed to represent adsorption strength. For a flat graphene system with one Ar atom, the *E*_ads_ is only 100 meV (Fig. 1b), such a value will be compensated by thermal fluctuations and interfacial energy, resulting in desorption. As the ripple curvature increases, *E*_ads_ progressively increases also. When the curvature reaches 0.116, *E*_ads_ rises to 171 meV, indicating enhanced adsorption. This trend holds with more adsorbed atoms (Fig. S1a), confirming curvature-dependent behaviour. Furthermore, *E*_ads_ increases as the molecular weight of the adsorbed gas also increases, i.e. the stable adsorption of Xe atoms on rippled graphene occurs more readily than that of He.

We experimentally utilize three different approaches to adsorb noble gases at RT. The adsorption process involves a weakly ionized plasma comprising electrons along with polarized and ionized noble gas species. Alternative methods can also yield equivalent adsorption results. Critically, those techniques ensure complete preservation of the integrity of the graphene lattice (details in Methods section). The kinetic energy of the activated gas atoms may intensify the ripple depths of graphene, thereby achieving stable adsorption. First, scanning tunnelling microscope (STM) imaging of Cu(111)\monolayer (1 L) graphene (Gra) film with adsorbed Xe atoms (ads-Xe) is performed under ultra-high vacuum. Figure 1e shows a typical large-scale STM image of ads-Xe on Cu(111)\Gra after a short period of annealing at 90°C. Unlike the well-defined Cu atomic steps, aggregates of ads-Xe form irregular domains with different thicknesses. While zooming-in on an ads-Xe domain, an obviously close packed structure of crystalized Xe is observed under 2 V bias voltage (Fig. 1c). The lateral distance between neighbouring Xe atoms is ∼6.8 Å (Fig. 1g, red line). When decreasing the bias voltage, the lattice arrangement of Xe undergoes different deformations, while the underlying graphene honeycomb lattice becomes increasingly discernible (Fig. 1d and Fig. S1b–f). Additionally, the ads-Xe atoms appear to keep strict alignment with the underlying lattice orientation of the graphene. The structural symmetry of ads-Xe emerges from 6-fold into 2-fold. The lattice spacing of graphene is measured to remain at ∼2.4 Å, but the aligned distance for ads-Xe is reduced to ∼5.9 Å (Fig. 1g green and blue lines). The ads-Xe can be completely removed via 350°C annealing, leaving the defect-free hexagonal lattices of graphene (Fig. 1f), indicating non-destructive adsorption-desorption processes.

The elemental identification of ads-Xe is performed with X-ray photoelectron spectroscopy (XPS), and the distinct characteristic peaks of Xe 3*d*_3/2_ and 3*d*_5/2_ core levels appear at 682.5 and 669.8 eV, respectively (Fig. 1h and Fig. S2). After subsequent desorption, the Xe-related spectral features vanish completely.

Figure 1i and j display the STM images of Cu(111)\Gra with partially adsorbed Ar atoms (ads-Ar) and partially adsorbed He atoms (ads-He), respectively. They are obtained following short-time annealing at 180°C. It is worth noting that the ads-Ar and ads-He inside the irregular domains usually present a dimer configuration with a periodical array, with the lateral distances of neighbouring Ar and He atoms within a dimer being ∼300 pm and ∼230 pm, respectively.

Moreover, scanning tunnelling spectroscopy (STS) of ads-Xe and ads-Ar graphene are further collected (Fig. S3). The domain regions of ads-Xe and ads-Ar exhibit distinct shoulder peaks, which is similar to the STS shoulder peak of Xe or Kr adsorbed on Au(111) at extremely low temperature [30]. The peak positions systematically shift with the annealing temperature, and there is also a small but clear bandgap of ∼80 meV in ads-Xe graphene.

### Noble gases adsorbed on suspending graphene

**Figure 2.**
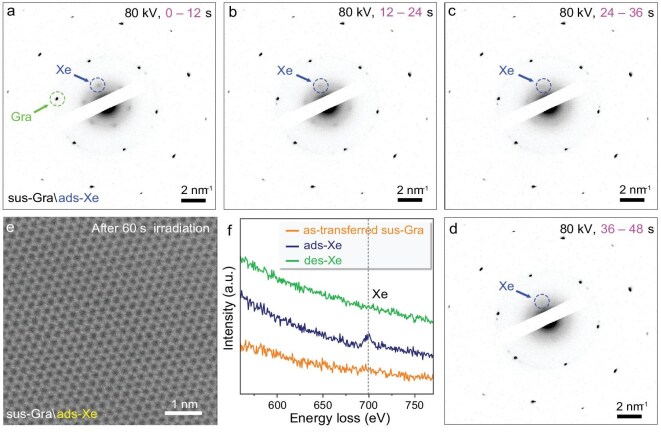
STEM observations of ads-Xe on suspending graphene. (a, d) Typical SAED patterns of sus-Gra with ads-Xe; the labelled green and blue dash circles originate from graphene and ads-Xe, respectively. The SAED patterns in (a, d) are successively captured for 12 s, and SAED patterns from ads-Xe disappear after ∼36 s e-beam irradiation. The sixfold symmetrical ads-Xe patterns correspond to the in-plane lattice constant of 5.3 ± 0.2 Å. (e) Typical STEM image of sus-Gra with ads-Xe after 60 s e-beam irradiation showing there are only perfect graphene lattices remaining. (f) EELS spectra of as-transferred sus-Gra, ads-Xe, and des-Xe graphene. A distinct Xe characteristic peak located at ∼699 eV is clearly observed in the ads-Xe graphene.

Figure 2a–d shows the selected area electron diffraction (SAED) of suspending graphene (sus-Gra) films with ads-Xe at different collection stages. There is an additional set of hexagonal symmetrical SAED patterns that have arisen besides graphene, and these extra patterns are attributed to the crystallized ads-Xe. For these measurements, we utilize a very low dose rate of ∼0.1 pA/μm^2^ and a 12-s collection duration, but these patterns are still gradually weakened with each measurement interval. After only 48 s exposure, the extra SAED pattern of ads-Xe crystals completely disappears, indicating the adsorbed Xe atoms are easily desorbed by the 80 kV electron beam. Following complete desorption, a perfect hexagonal graphene lattice without any crystalline defects is obtained (Fig. 2e), indicating the non-destructive feature of the adsorption-desorption process. Notably, the observed SAED pattern for adsorbed Xe atoms on graphene at RT corresponds to the lattice constant of 5.3 ± 0.2 Å, which is smaller than the value obtained by STM measured at 77 K. Additional SAED patterns collected at random locations further show that the lattice orientations of ads-Xe always align with the underlying graphene (Fig. S4).

To further confirm the existence of ads-Xe on sus-Gra, electron energy loss spectroscopy (EELS) equipped in the scanning transmission electron microscope is performed under the mildest conditions of 60 kV and the lowest dose rate of 0.025 pA/μm^2^. After cumulative collection over 3600 s, a discernible Xe characteristic peak at ∼699 eV is detected (Fig. 2f). However, even under the mildest adjustable conditions, we are still unable to detect the characteristic peaks from ads-Kr, ads-Ar, or ads-He, probably due to their easier desorption compared with ads-Xe.

### Stability of the ripple-assisted adsorption

**Figure 3.**
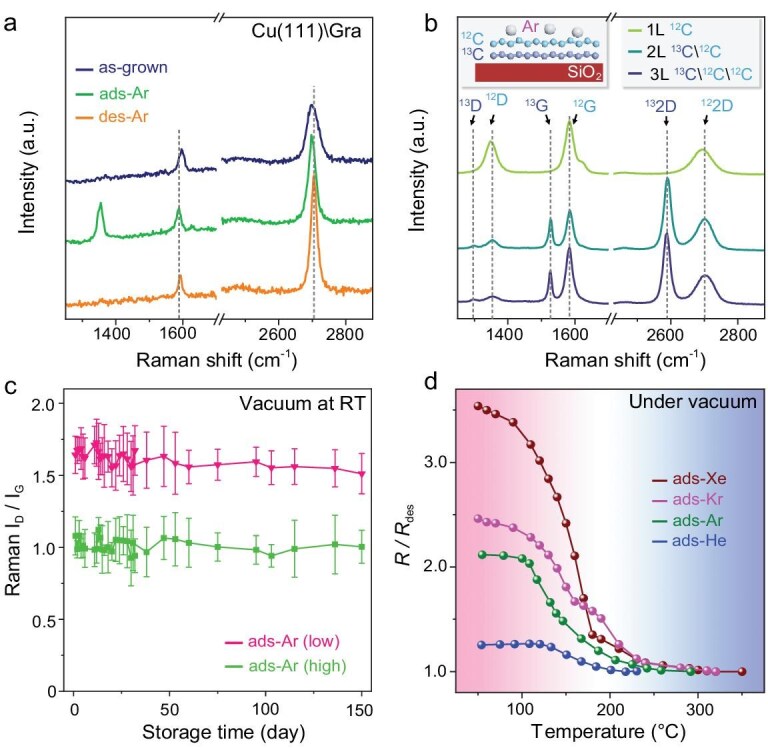
Structural changes of graphene upon adsorption of noble gases. (a) Raman spectra of as-grown, ads-Ar, and des-Ar graphene on Cu(111). The ads-Ar induce the *sp*^3^ phase transition (D peak) in graphene and the rippled deformation can be completely recovered. (b) Raman spectra of graphene with different layer numbers adsorbing Ar. The 2 L and 3 L graphenes consist of stacked ^12^C and isotopically labelled ^13^C graphene films (^13^C\^12^C for 2 L and ^13^C\^12^C\^12^C for 3 L). Inset is a schematic of the SiO_2_\^13^C\^12^C with ads-Ar. (c) Stability of I_D_/I_G_ in prolonging storage duration. Graphene with different I_D_/I_G_ remains nearly unchanged at RT within 150 d. (d) *In situ* variable-temperature *R*/*R*_des_ of graphene films with adsorbed Xe, Kr, Ar, and He with the same ripple deformation. The temperature for desorbing Xe is highest and that for He is lowest. Generally, the *R*/*R*_des_ starts to descend from 120°C and recovers to its normal state above 350°C.

The stability of adsorbed noble gas atomic crystals is monitored by the property changes of graphene. Figure 3a plots typical Raman spectra of the as-grown graphene film on Cu(111), ads-Ar, and des-Ar graphene, while Raman is highly sensitive to graphene lattice deformation the intensity ratio of the D to G peak (I_D_/I_G_) can be utilized as an indicator of non-*sp*^2^ hybridization [31–34]. The as-grown graphene is defect-free, and an obvious D peak (∼1350 cm^−1^) appears for ads-Ar. The D peak can be fully eliminated for des-Ar by thermal annealing. This adsorption-induced D peak, as well as its removal, is also applicable to other adsorbed noble gases (Fig. S5a). Given the chemical inertness of Ar atoms, the appearance of D peaks can be more plausibly attributed to the out-of-plane displacement of carbon atoms rather than the formation of C-Ar bonds, although the latter possibility cannot be ruled out, and the disappearance of the D peak corresponds to complete desorption, which behaves differently between the origins of edges, atomic vacancies, and C-H bonds [33,35]. Along with the adsorption, both the G and 2D peaks exhibit a minor redshift, primarily due to the enhanced tensile strain (Fig. S5b–d).

Figure 3b plots the Raman spectra of transferred graphene (1−3 layers) on Si\SiO_2_ after adsorbing Ar at RT. The bilayer (2 L) and triple-layer (3 L) graphene consists of stacked ^12^C and isotopically labelled ^13^C graphene films, named as ^13^C\^12^C and ^13^C\^12^C\^12^C, respectively. Under the same adsorption conditions, 1 L ^12^C graphene exhibits the highest ripple deformation, with an I_D_/I_G_ ratio of ∼0.61. The I_D_/I_G_ ratio decreases as the number of layers increases, and the ^12^I_D_/^12^I_G_ ratio decreases to ∼0.22 for 2 L graphene, while the ^13^I_D_/^13^I_G_ ratio is ∼0.09 for 3 L graphene. Noting that, the I_D_/I_G_ of ^12^C and ^13^C in different layer order both show reduction, indicating that rippled deformations are formed in all the layers and progressively diminish as the layer depth increases. These ripple-induced D peaks can also be completely recovered by thermal annealing (Fig. S5e).

Additional studies on fewer exfoliated layers of graphene (1–4 layers) further confirm that the I_D_/I_G_ ratio systematically weakens as the number of layers increase (Fig. S5f–g). Moreover, the adsorption process is also influenced by the substrate on which graphene is contacted. Under identical adsorption conditions, graphene on a substrate demonstrates a higher I_D_/I_G_ ratio compared to suspended graphene (Fig. S5h–j). A longer adsorption duration cannot enhance ripple deformation once reaching its maximum, and they can be fully recovered after desorption. Furthermore, the adsorbed gas atoms on graphene are uniformly distributed across the whole wafer and remain highly stable, remaining nearly unchanged even under vacuum for 5 months at RT (Fig. 3c and Fig. S6).

Figure 3d plots the temperature-dependent normalized resistance (*R*/*R*_des_) of graphene with adsorbed Xe, Kr, Ar, and He under vacuum (<10^−2^ Pa), where the *R*_des_ represents the longitudinal resistance (*R*_xx_) after complete desorption. The desorption processes for all the adsorbed noble gases begin at ∼120°C, with the temperatures for complete desorption of Xe, Kr, Ar, and He being ∼350, ∼320, ∼250, and ∼230°C, respectively. Notably, despite exhibiting a similar degree of ripple deformation (Fig. S7a), these graphene films with adsorbed Xe, Kr, Ar, and He exhibit distinct resistivity. This disparity likely results from differential interactions between adsorbed gas species and the deformed carbon atoms. We further fit the activation energies for gas desorption processes via the Arrhenius law *R* = A‧exp(-*E*_a_/*k*_B_T), where *R* is the relative resistance, *k*_B_ is the Boltzmann constant, A is pre-exponential factor, T is the measured temperature, and *E*_a_ is the activation energy for desorption. The derived values for desorbing Xe, Kr, Ar, and He are 207 meV, 106 meV, 83 meV, and 48 meV, respectively (Fig. S7b and c).

In contrast, all the adsorbed noble gases start to desorb at ∼110°C under AP, which is basically consistent with the desorption under vacuum (Fig. S7d and f). The critical temperatures for complete desorption are independent of the ripple deformation degree for a given noble gas (Fig. S7g), indicating the primary dependence on the adsorbate-adsorbent interaction rather than ripple deformation.

### Adsorption-induced physical property changes

**Figure 4.**
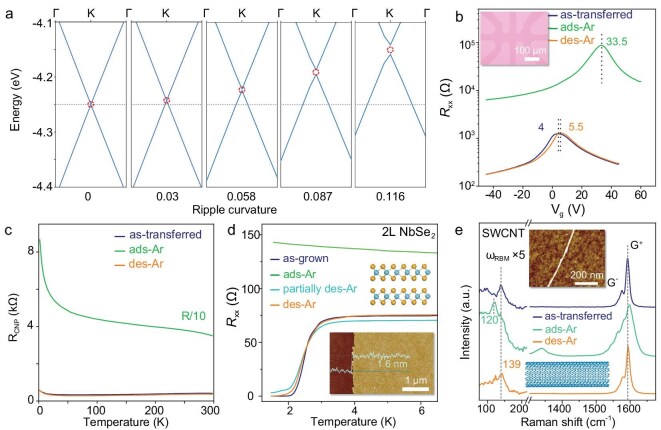
Physical property changes of 2D materials with adsorbed noble gases. (a) Theoretically calculated band structures of graphene with different ripple curvatures; the curvatures from left to right are 0, 0.03, 0.058, 0.087, and 0.116, respectively. (b) Typical electrical transport of as-transferred, ads-Ar, and des-Ar graphene films on Si\SiO_2_. Inset is the optical image of the graphene Hall bar. (c) Variable-temperature *R*_CNP_ of as-transferred, ads-Ar, and des-Ar graphene films. (d) Variable-temperature *R*_xx_ of as-grown, ads-Ar, and des-Ar 2 L NbSe_2_ films. Top inset is the atomic model of 2H phased 2 L NbSe_2_, and bottom inset is the typical AFM image of 2 L NbSe_2_ film on sapphire. (e) Raman spectra of as-transferred, ads-Ar, and des-Ar SWCNT. Inset is the corresponding AFM of as-transferred SWCNT on Si\SiO_2_.

Figure 4a shows the theoretically calculated band structure of rippled graphene with ads-Ar. As the ripple curvature increases, the Fermi level of graphene shifts from −4.25 eV to −4.15 eV, indicating a gradually enhanced *p*-type doping introduced by symmetry breaking. Concurrently, the rippled lattices also induce a bandgap opening of ∼20 meV when the ripple curvature reaches 0.116. Additionally, the Fermi level of rippled graphene remains unchanged regardless of the presence of Ar atoms, i.e. the change of band structure mainly results from the rippled lattice rather than adsorbed Ar (Fig. S8).

Figure 4b plots the *R*_xx_ of as-transferred, ads-Ar and des-Ar graphene films on Si\SiO_2_ at different gate voltage (*V*_g_). The gate voltage at the charge neutrality point (*V*_CNP_) of as-transferred graphene shifts from 4 to 33.5 V after adsorbing Ar, indicating *p*-type doping [36,37]. The *V*_CNP_ for des-Ar graphene recovers to 5.5 V. Moreover, the *R*_xx_ of ads-Ar graphene is nearly two orders of magnitude larger than that of the as-transferred film, which is comparable to hydrogenated and fluorinated graphene with approximate Raman I_D_/I_G_ ratio [33,38], and much larger than graphene with adsorbed polar NH_3_ or NO_2_ gases [39]. The *R*_xx_ and transport characteristics of des-Ar graphene are completely recovered.

Figure 4c further compares the variable-temperature *R*_xx_ at *V*_CNP_ (*R*_CNP_) for as-transferred, ads-Ar, and des-Ar graphene. Ads-Ar graphene behave like a typical semiconductor regardless of the different doping levels. The derived transport bandgap of ads-Ar graphene is fitted as ∼7.4 meV, indicating the bandgap opening (Fig. S9).

Figure 4d plots the variable-temperature *R*_xx_ of as-grown, ads-Ar, partially des-Ar, and des-Ar NbSe_2_ films, where the superconductivity for NbSe_2_ is highly sensitive to the structural change [40]. As-grown 2 L NbSe_2_ films show a superconducting transition critical temperature (*T*_c_) of 2.25 K. In contrast, the ads-Ar NbSe_2_ behaves as a typical semiconductor with nearly doubled *R*_xx_, and the corresponding Raman characteristic peaks are also changed (Fig. S10a). Partial des-Ar and des-Ar NbSe_2_ gradually recovers superconducting behaviour with a *T*_c_ of 1.95 K and 2.15 K, respectively. Similarly, the photoluminescence (PL) of 1 L MoS_2_ also changes after Ar adsorption, and the desorption process can also gradually recover the characteristics of a semiconductor [41], with slightly changed Raman peaks (Fig. S10b–d).

Analysis of individual single-walled carbon nanotubes (SWCNTs) [42] is also performed, and the Raman radial breathing mode (RBM) of SWCNTs is sensitive to structural deformation [43]. Figure 4e plots the *ex situ* Raman spectra of as-transferred, ads-Ar and des-Ar individual SWCNTs on a Si\SiO_2_ substrate. The as-transferred SWCNT is almost defect-free, and the *ω*_RBM_ is located at 139 cm^−1^. After Ar adsorption, the D band appears and additional sub-peaks arise in the G peak region for the same position of SWCNT. More critically, the *ω*_RBM_ redshifts to 120 cm^−1^. After desorption, the shapes of D and G peaks along with the *ω*_RBM_ all return to the initial state, confirming the recovery of the radial structural deformation.

## CONCLUSIONS

We propose a ripple-assisted adsorption mechanism to realize the stable adsorption of crystallized noble gases on graphene and other layered materials at RT, where the enhanced rippled structure reduces the adsorption energy. The adsorbed noble gas atoms on graphene exhibit packed atomic crystals with periodic arrangement, and the adsorption maintains stable even for half a year at RT. They can be desorbed at elevated temperature starting from 110°C, while the recovered graphene lattice remains completely intact without any structural damage. The elemental characteristics of adsorbed noble gases on graphene are comprehensively confirmed. Adsorption-induced ripple deformation modifies the structural and physical properties of graphene, NbSe_2_, MoS_2_, and SWCNT, where their electrical transport, superconductivity, PL, and Raman RBM are hugely affected. These property changes can be completely recovered after desorption. The ripple-assisted adsorption is totally different from conventional physisorption and chemisorption theory, and hence we believe this adsorption for different gases, especially nonpolar gases, will accelerate the process of gas storage and separation technologies, as well as facilitate their applications in catalysis, surface modification, and other related fields.

## Supplementary Material

nwaf506_Supplemental_File
