# Supplementary material for: Ripple-assisted adsorption of noble gases on graphene at room temperature
Source: Natl Sci Rev. 2025 Nov 14;13(1):nwaf506. doi: 10.1093/nsr/nwaf506 (PMC12805829; doi:10.1093/nsr/nwaf506)
Supplement: nwaf506_Supplemental_File [file nwaf506_supplemental_file.pdf]

Supporting Information for

Ripple-assisted adsorption of noble gases on graphene at room temperature

Weilin Liu[1,†], Xianlei Huang[1,†], Li-Guo Dou[1,†], Qianglong Fang[2,†], Ang Li[3,†], Guowen Yuan[1,*], Yongjie Xu[1], Zhenjia Zhou[1], Jun Li[1], Yu Jiang[1], Zichong Huang[1], Zihao Fu[1], Peng-Xiang Hou[4], Chang Liu[4], Jinlan Wang[2,5], Wu Zhou[3*], Ming-Gang Ju[2,*], Shao-Chun Li[1,6,*], Hui-Ming Cheng[4,7] and Libo Gao[1,8,*]

[1]National Laboratory of Solid State Microstructures, Jiangsu Key Laboratory for Nanotechnology, Jiangsu Physical Science Research Center, School of Physics, Nanjing University, Nanjing 210093, China;

[2]Key Laboratory of Quantum Materials and Devices of Ministry of Education, School of Physics, Southeast University, Nanjing 210096, China;

[3]School of Physical Sciences, University of Chinese Academy of Sciences, Beijing 101408, China;

[4]Shenyang National Laboratory for Materials Sciences, Institute of Metal Research, Chinese Academy of Sciences, Shenyang 110016, China;

[5]Suzhou Laboratory, Suzhou 215000, China;

[6]Hefei National Laboratory, Hefei 230026, China;

[7]Institute of Technology for Carbon Neutrality, Shenzhen Institute of Advanced Technology, Chinese Academy of Sciences, Shenzhen 518055, China;

[8]State Key Laboratory of Chemo/Biosensing and Chemometrics, Key Laboratory for Micro-Nano Physics and Technology of Hunan Province, College of Materials Science and Engineering, Hunan University, Changsha 410082, China

***Corresponding authors**. E-mails: gwyuan@nju.edu.cn; wuzhou@ucas.ac.cn; juming@seu.edu.cn; scli@nju.edu.cn; lbgao@nju.edu.cn

[†]Equally contributed to this work.

EXPERIMENTAL AND METHODS

Sample preparation

Exfoliated graphene flakes. Few-layer graphene flakes are exfoliated onto Si substrates with 285 nm thick oxide (Si\SiO_2) by a traditional micromechanical cleavage method from kish graphite. Then, all the flakes are annealed under high vacuum (<10^{-5} Pa) at 350 °C for 30 min to remove the polymer residues.

CVD graphene films. Large-area ultra-flat graphene films are grown by the proton-assisted chemical vapour deposition method as reported previously[36]. The typical growth parameters are as follows: sputtered 800 nm thick Cu-Ni(111) films on c-plane sapphire as substrates, growth temperature of 650 °C, pressure of 6 Pa, CH_4/H_2 ratio of 1:20, plasma power of 15 W and growth time of 5 min. The growth parameters for traditional CVD grown graphene films are as follows: sputtered 800 nm thick Cu-Ni(111) films on c-plane sapphire as substrates, growth temperature of 1050 °C, CH_4/H_2/Ar ratio of 0.1:10:500 under AP, growth time of 10 min. We use Cu-Ni alloy (90% Cu + 10% Ni) instead of Cu in this study to avoid sublimation at 1050 °C or vacuum annealing at 650 °C. To simplify the writing, we use Cu(111) instead of Cu-Ni(111) alloy in the main text.

CVD $NbSe_2$ films. 2L $NbSe_2$ are grown by the two-step vapour deposition method as reported previously[40]. The typical sputter parameters are as follows: totally 1.0 nm thick Nb films, c-plane sapphire as sputtered substrate, substrate temperature of ~200 °C, deposition rate of ~0.2 Å/s and the constant chamber pressure of 10 Pa. The typical CVD growth parameters are as follows: heating Se powder (>99.9%) of 340 °C in zone I upstream, heating Nb films of 650 °C in zone II downstream, growth time of 15 min, the carrier gas of H_2/Ar (100:100) under AP. After the growth, the $NbSe_2$ films are annealed at 400 °C for 30 min in zone II to remove the redundant Se particles (zone I is not heated).

CVD MoS_2 grains. 1L MoS_2 grains are grown by the two-step vapour deposition as reported previously[40]. The sputter and CVD growth parameters are similar to those of $NbSe_2$ above. The sputtered Mo films are 0.7 nm, and the temperatures of heating S powder (>99.9%) and Mo film are 160 °C and 800 °C, respectively.

CVD SWCNT. An injection floating catalyst chemical vapor deposition (FC-CVD) is used for

growing the isolated SWCNT networks as reported previously[42]. The growth and precursor temperatures are 1100 and 83 °C with pure Ar atmosphere under AP, respectively. Then, the carrier gas of C_2H_4/H_2 ratio of 5:2 is introduced, along with the mixed solution containing 96 wt.% toluene, 3 wt.% ferrocene, and 1 wt.% thiophene injected into the reactor at a rate of 4 sccm through a syringe pump. The SWCNT films with different thicknesses are collected on porous cellulose filter membranes installed at the outlet of the flowing gases. The individual SWCNTs are transferred from the porous cellulose filter membranes onto the Si\SiO_2 substrates by simple pressing.

Transfer of CVD graphene films. After CVD growth, the sapphire\Cu(111)\graphene is spin-coated with double layers of polymethyl methacrylate (PMMA, first layer, 120k MW, 1 wt.% in ethyl lactate, 2000 rpm for 1 min; second layer, 996k MW, 4 wt.% in ethyl lactate, 2000 rpm for 1 min) and baked at 150 °C in air for 10 min. Then, 1 M $(NH_4)_2S_2O_8$ aqueous solution is used to etch the Cu(111). After cleaned with DI water thrice, the floating graphene\PMMA is pasted onto the target substrate, including Si\SiO_2 wafers and microgrids. Subsequently, substrate\graphene\PMMA is baked at 40 °C for 6 h, 80 °C for 30 min and 150 °C for 10 min in sequence. Finally, PMMA films are removed by acetone and the further proton-assisted cleaning process[37].

Characterizations

Optical microscope. All the optical images are captured by the optical microscope (Nikon Eclipse LV100ND).

Raman. Raman spectra are acquired using a WITec/alpha 300R confocal microscope with 532 nm laser under ambient conditions. The variable-temperature Raman measurements are performed in a temperature-controlled stage (Linkam THMS600), which is integrated in the Raman system. All the laser power is set below 2 mW to avoid heating.

AFM. AFM measurements are performed with a Bruker Dimension Fastscan system at tapping mode.

XPS. XPS is performed using a commercial PHI 5000 X-ray photoelectron spectrometer equipped with a monochromatized Al Kα radiation (hv = 1486.6 eV). The measurements are performed at RT under UHV with the X-ray power of 25 W. The X-ray spot size is approximately 100 × 100 μm^2, while the sampling analysis area is about 1350 × 700 μm^2. Peak fitting is performed using a mixed Lorentzian-Gaussian function.

STM and STS. The STM measurements of ads-Xe, ads-Ar, and ads-He on Cu(111)\Gra are performed under UHV at 77 K (USM 1500, Unisoku). The topographic images are measured under the constant current mode, and the differential conductance (d*I*/d*V*) spectra are taken using a standard lock-in techniques at 77 K (f=879 Hz, ΔV_{rms}=12 mV). Partially desorption process is performed to obtain the clear high-resolution imaging. The ads-Xe, ads-Ar, ads-He graphene in Fig. 1c, 1i and 1j are annealed under UHV at 90 °C for 1.5 h, 180 °C for 1.5 h, 140 °C for 12 h before STM measurement, respectively.

STEM. The STEM measurements are performed on a Nion U-HERMES100 microscope with an acceleration voltage of 60 kV under UHV. The convergence semi-angle is set to 32 mrad, and the e-beam current for imaging during the experiments is ~18 pA. The STEM-EELS measurements are performed using a collection semi-angle of 75 mrad, an energy dispersion of 0.9 eV per channel and a probe current of ~0.1 pA. The dose rate is further reduced by a large defocus probe (~5 μm). The EELS camera is a direct electron EELS detector (DECTRIS ELA) with single electron sensitivity, enabling the low dose rate measurement. The SAED patterns are collected in a JEOL 2100Plus TEM with an acceleration voltage of 80 kV.

Electrical properties. The electrical transport measurements and variable-temperature resistance measurements of graphene, and $NbSe_2$ films below 300 K are performed in a ^4He cryostat with a superconducting magnet (Oxford Teslatron 8 T), and the standard four-terminal method is adopted with a lock-in amplifier (Stanford SR830) at a frequency of 3.67 Hz and the bias current of 100 nA. The variable-temperature resistance measurements above 300 K of graphene films are performed in a heating stage under vacuum (<10^{-2} Pa) with the bias current of 1 μA (Keithley 2450) and monitored voltage (Keithley 2182A). For superconductivity, the T_c temperature is calibrated as the sheet resistance drops to 10% of its normal state.

Controllable adsorption of gas molecules

Three methods are used to inject net electrons in graphene. The specific operations are as follows:

Charge injection in SEM. The negative charges (electron) are controllably emitted from electron gun of environmental scanning electron microscope (SEM, FEI Quanta 200). To avoid high-energy electrons irradiate graphene directly, electrons are chosen to radiate the Cu(111) region of Cu(111)\Gra sample and soldered indium of SiO_2\Gra samples. The Ar gas flow into SEM by a valve. Typical

adsorption parameters are as follows: constant pressure of 10 Pa, accelerating voltage of 30 kV, emission current of ~80 pA, adsorption time of 30 min, at RT.

Charge injection in customized instrument. Customized instrument is designed to mimic the charge injection process in SEM. The negative charges (electron) are controllably emitted from an electrostatic generator (JTStar, JT206B), which consists of several tungsten tips and a high voltage power supply (0 to -20 kV). The emitted electrons are collected at a copper plate and transmitted to the target samples via a copper wire. The injected electron density is monitored by a high precision ampere meter (KangWei, KV-MCM02503) calibrated by a source meter (Keithley 6430). To avoid the glow discharge, the charging density by election injection is usually <10 nA/mm^2. The typical adsorption parameters are as follows: gas pressure of 133 Pa (1 Torr), charging current density of 0.55 nA/mm^2. The adsorption degree is modified by the charging time.

Plasma treatment. The controllable plasma of different gas is generated in a customized inductively coupled plasma (ICP) system, and typical treatment parameters are as follows: flow rate of 120 sccm, constant pressure of 6 Pa, plasma power of 10 W, treatment time of 10 – 120 s, adsorption temperature of 35 °C.

Desorption by thermal annealing. For *ex situ* desorption, all the treatments are performed in a high vacuum (<10^{-5} Pa), and their thermal annealing conditions are as follows: graphene, 350 °C, 30 min; MoS_2, 350 °C, 30 min; $NbSe_2$, 150 °C, 30 min for partial desorption, 170 °C, 30 min for complete desorption; SWCNT, 350 °C, 30 min.

Theoretical calculation for total adsorption energy

A graphene sheet with a diameter of 12.4 Å passivated by hydrogen atoms is chosen to model the adsorption of noble gas on the rippled graphene surface. Geometric optimization and energy calculations are performed using the Gaussian 16 program package at the M06-2X/SDD level of theory for noble gases, including He, Ne, Ar, Kr and Xe. This level of theory is selected to fully consider the relativistic effects. The atoms in the upper layer and the bottom layer are fixed, while the atoms in the middle layer relax freely. The adsorption energy (E_{ads}) is defined as:

$$E_{ads} = E_{Gra+gas} - E_{Gra} - E_{gas}$$

Where $E_{Gra+gas}$, E_{Gra} and E_{gas} are the total energies of rippled graphene adsorbed with noble gas, single

ripple graphene sheet and one noble gas, respectively.

The band structures of graphene with different ripple curvature are calculated based on the density functional theory (DFT) implemented in the Vienna Ab initio Simulation Package (VASP) with the projector-augmented wave method. The GGA-PBE function is used to describe the exchange-correlation functionals. The plane-wave cutoff energy is set to 450 eV, and the force converged to 0.01 eV/Å. Dipole correction is employed to eliminate the potential errors, and the van der Waals interaction between graphene and Ar atom is included by DFT-D3.

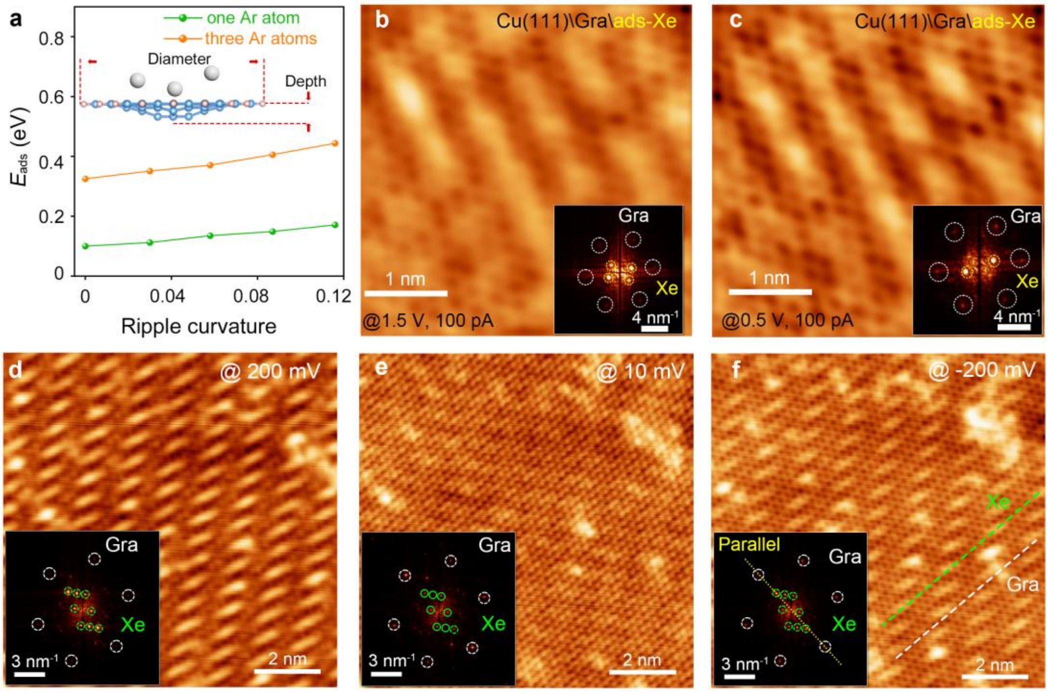

Figure S1. Additional STM images of ads-Xe graphene on Cu(111). **a** Theoretical calculated E_{ads} of graphene with different ripple curvature adsorbing noble gas atoms. Inset illustrates the side view of rippled graphene (96 carbon atoms) adsorbing three Ar atoms. **b**-**c**, *in situ* STM images of ads-Xe graphene measured at different bias voltage share the same position as those in main Fig. 1d and 1e. Insets are the corresponding FFT patterns. **d**-**f**, *in situ* STM images of ads-Xe graphene measured with different bias voltage. As changing the bias, the surface morphologies show obviously different, where the Xe atomic crystals are clearer when applying higher bias voltage and graphene lattice becomes clearer when applying lower bias voltage. Insets are the corresponding FFT patterns. The Xe atomic crystal arrangements exhibit a consistent orientation with the graphene lattice's crystalline structure, as indicated by the dashed line in panel (**f**).

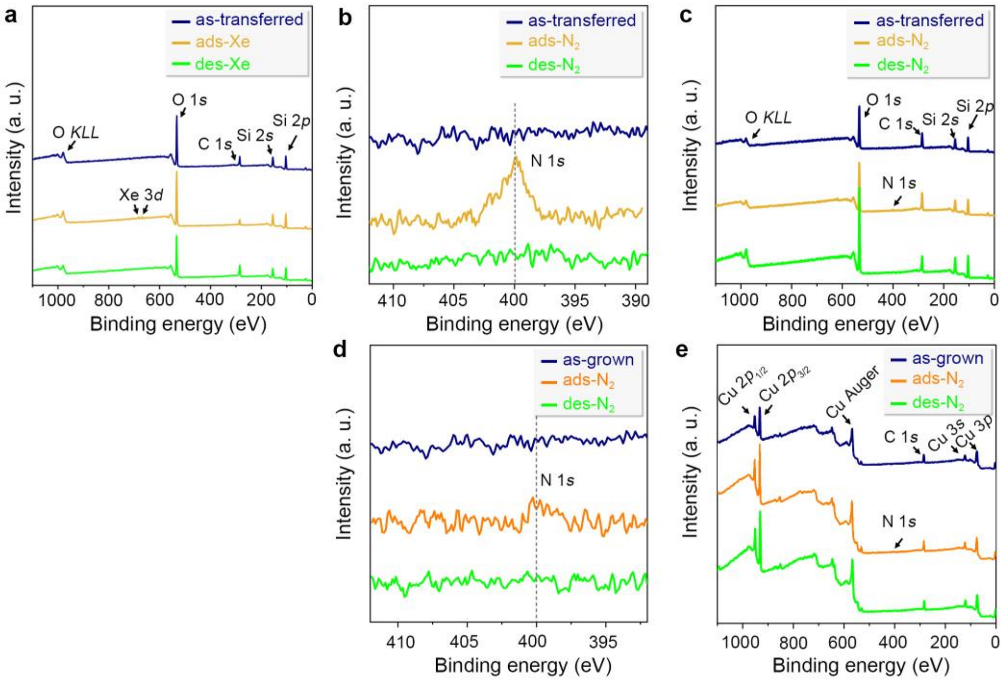

Figure S2. Additional XPS data for ads-Xe and ads-N_2 graphene. **a**, Corresponding survey XPS spectra of as-transferred, ads-Xe and des-Xe graphene on Si\SiO$_2$. **b**, Fine XPS spectra of as-transferred graphene, ads-N_2 graphene and des-N_2 graphene (vacuum annealing at 300 °C) on Si\SiO$_2$. The apparent N characteristic of N-N core level is located at ~400 eV for ads-N_2 graphene. **c**, Corresponding survey XPS spectra of as-transferred graphene, ads-N_2 and des-N_2 graphene on Si\SiO$_2$. **d**, Fine XPS spectra of as-grown, ads-N_2 and des-N_2 graphene on Cu(111) under the same adsorption condition with **b**. The apparent N characteristic of N-N core level is located at ~400 eV for ads-N_2 graphene. **f**, Corresponding survey XPS spectra of as-grown, ads-N_2 and des-N_2 graphene on Cu(111).

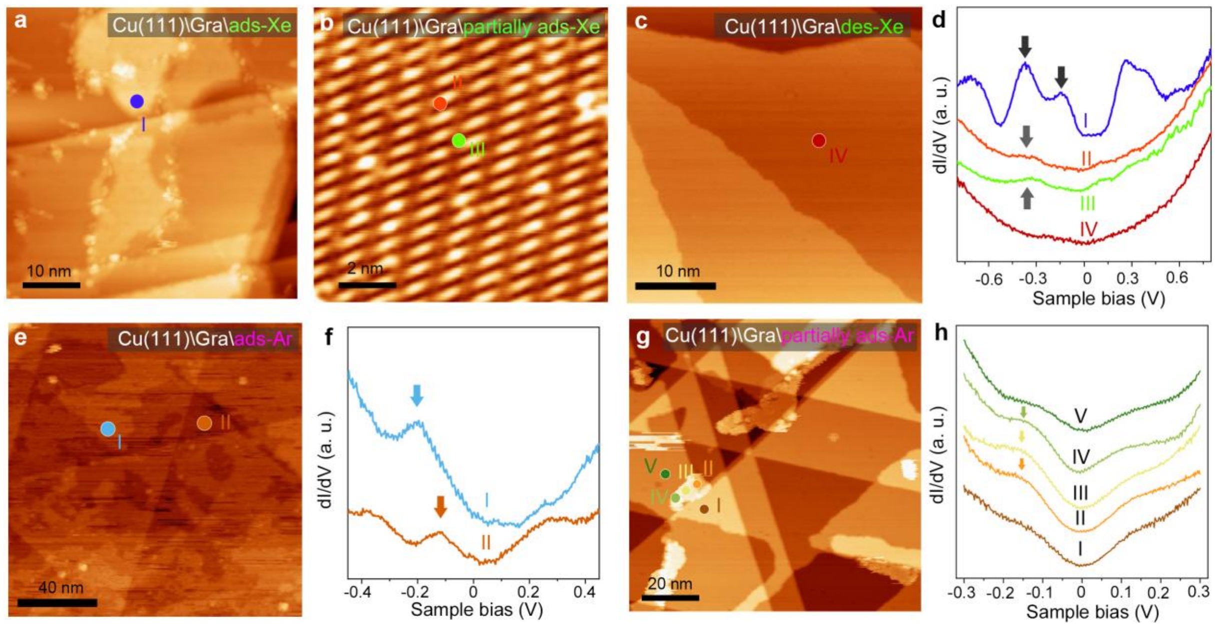

Figure S3. Additional STM images and STS spectra of ads-Xe and ads-Ar graphene on Cu(111). **a**, Typical STM topological image of ads-Xe graphene. **b**, Typical STM topological image of partially ads-Xe graphene after 180 °C annealing, part of adsorbed Xe remains. **c**, Typical STM topological image of des0Xe graphene after 350 °C annealing, no adsorbed Xe left. **d**, Corresponding STS spectra from the labelled regions in **a**-**c**. There are shows shoulder peaks (arrow-marked) and a bandgap opening for ads-Xe graphene. **e**, Typical STM topological image of ads-Ar graphene. **f**, Corresponding STS spectra from the labelled regions in **e**. There are shows shoulder peaks (arrow-marked) and a bandgap opening for ads-Ar graphene. **g**, Typical STM topological image of partially ads-Ar graphene after 180 °C annealing, part of adsorbed Ar remains. **h**, Corresponding STS spectra from the labelled regions in **g**. The shoulder peak persists in ads-Ar graphene with the changed peak position and intensity.

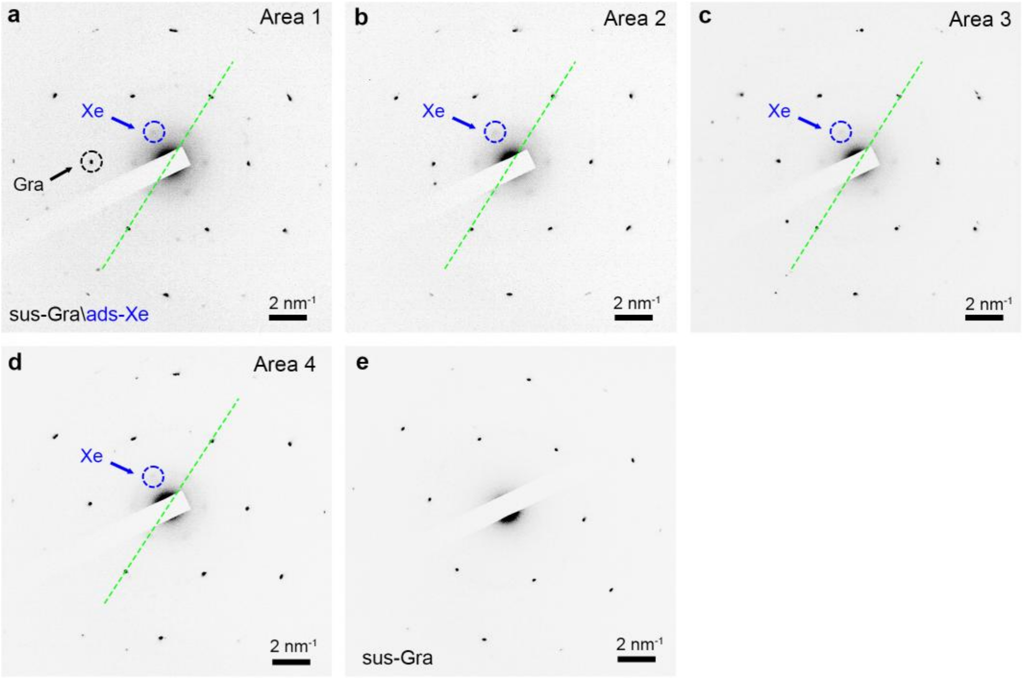

Figure S4. Additional SAED patterns of ads-Xe on suspending graphene. **a-d**, Additional SAED patterns of graphene with adsorbed Xe atoms at different locations. The black and blue dashed circles labelled in diffraction patterns represent graphene and crystallized Xe, respectively. The dashed lines indicate that the diffraction spots of crystallized Xe always align with the diffraction spots of graphene. **e**, SAED pattern of pure suspending graphene.

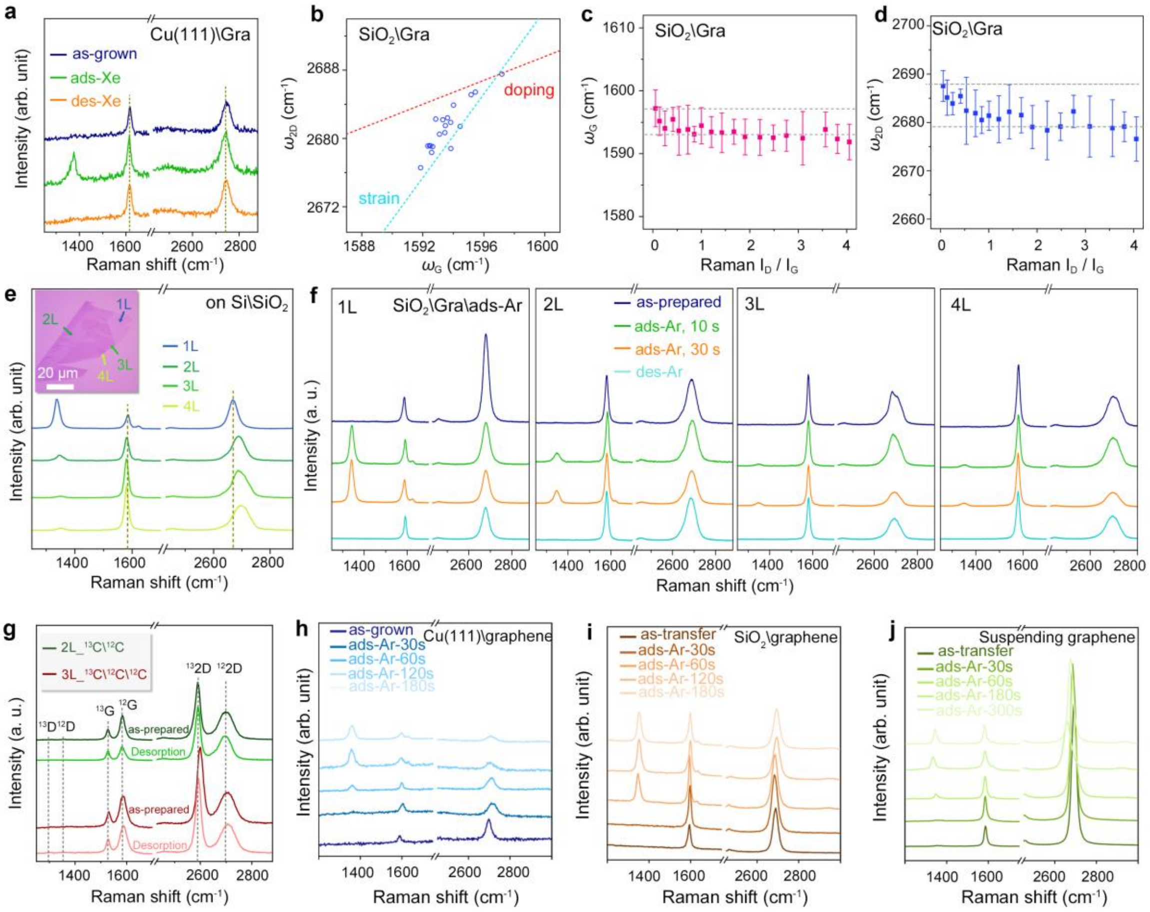

Figure S5. Additional Raman data of ads-Xe and ads-Ar graphene. **a**, Raman spectra of as-grown, ads-Xe and des-Xe graphene on Cu(111). The adsorbed Xe atoms induce the sp^3 phase transition (D peak) and the rippled deformation of graphene can be completely recovered. **b**, Relationship between ω_G and ω_{2D}, the dash lines are guides to the strain and doping induced frequency shifts, indicating the tension strains are introduced when graphene adsorbing Ar. **c**, Relationship between the frequency of G peak (ω_G) and I_D/I_G for ads-Ar graphene, the ω_G shows slight redshift of ~5 cm^{-1}. **d**, Relationship between the frequency of 2D band (ω_{2D}) and I_D/I_G for ads-Ar graphene, the ω_{2D} shows slight redshift of ~8 cm^{-1}. **e**, Raman spectra of graphene with different layer number adsorbed by Ar atoms. Inset is the corresponding optical image of exfoliated graphene flakes on Si\$SiO_2$. **f**, Raman spectra of exfoliated graphene varying layer numbers on Si\$SiO_2$ after adsorbing Ar through weak ICP with different adsorption time, from left to right is 1L, 2L, 3L and 4L. The I_D/I_G are rapidly reduced as increasing the layer number of graphene, indicating the ripple deformation is restricted for thick layer structures. **g**, Raman spectra of as-transferred and des-Ar 2L & 3L graphene stacked by the top ^{12}C and bottom isotopic ^{13}C. The rippled deformation of graphene can be completely recovered. **h-j.** Raman spectra of graphene on Cu(111), Si\$SiO_2$ and suspending graphene after adsorbing Ar through weak ICP with different adsorption time. As the adsorption time increasing, the change rate of the I_D/I_G of graphene on Cu(111) is the fastest, while that of the suspended graphene film is the slowest.

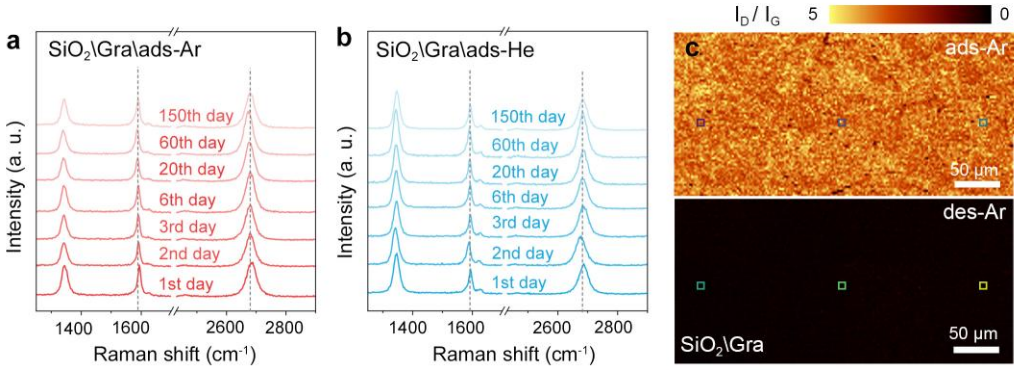

Figure S6. Additional Raman data of adsorption stability and uniformity. **a-b**, Typical Raman spectra of ads-Ar graphene stored for different durations under vacuum, these data correspond to the main Fig. 3c, indicating the adsorption are very stable at RT. **c**, Raman I_D/I_G mapping of ads-Ar and des-Ar graphene films. The I_D/I_G are both homogenous across the large area.

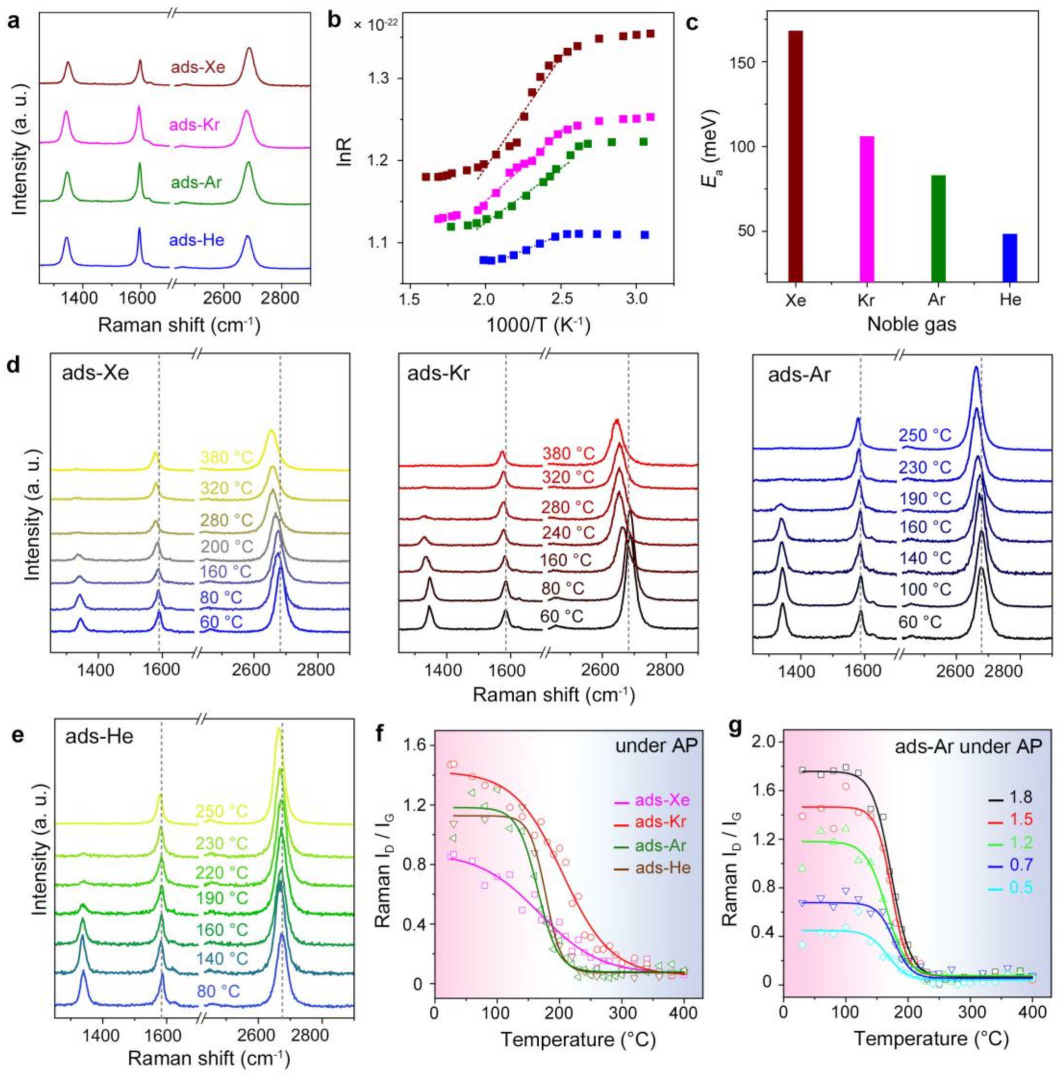

Figure S7. Additional Raman data of ads-gas or des-gas graphene on Si\SiO$_2$. a, Corresponding Raman spectra of ads-Xe, ads-Kr, ads-Ar and ads-He graphene in the main Fig. 3d, they have the similar I_D/I_G but the different resistances. **b**, The resistance of gas-adsorbed graphene at different annealing temperatures. Coloured dash lines are used to denote the fitted slopes in regions of significant resistance variation. **c**, Corresponding activation energies (E_a) for gas desorption from graphene, as derived from the data in (**b**). **d**-**e**, *in situ* Raman spectra of ads-Xe, ads-Kr, ads-Ar and ads-He graphene films as elevating temperature, all their D peaks completely disappear at above 350 °C. **f**, Extracted Raman I_D/I_G evolution of ads-Xe, ads-Kr, ads-Ar and ads-He graphene films as elevating temperature under AP. Their non-*sp*^{2} phased lattices start to be reduced from 100–150 °C, and all the lattices can be completely recovered above 350 °C. The noble gas with larger atomic weight has a higher temperature for complete desorption. **g**, Extracted Raman I_D/I_G evolution of ads-Ar graphene with different ripple deformations as elevating temperature under AP. Although the ripple deformations are different, the desorption starting and finishing temperature keep the consistent.

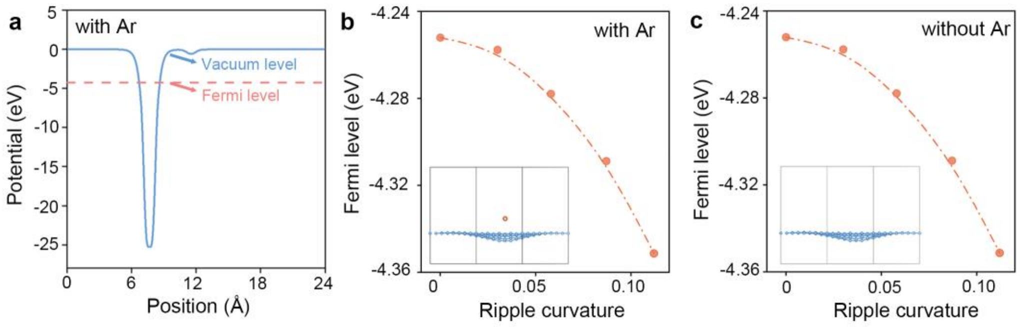

Figure S8. Additional data for calculated band structure of rippled graphene. **a**, Theoretical calculated potential energy of rippled graphene, where the vacuum level is shown as reference and initial value is set to 0. **b**, Calculated Fermi level of rippled graphene with adsorbed Ar as the function of ripple curvature, inset is the typical side view of one adsorbed Ar atom on rippled graphene. **c**, Calculated Fermi level of pure graphene as the function of ripple curvature, inset is the typical side view of rippled graphene.

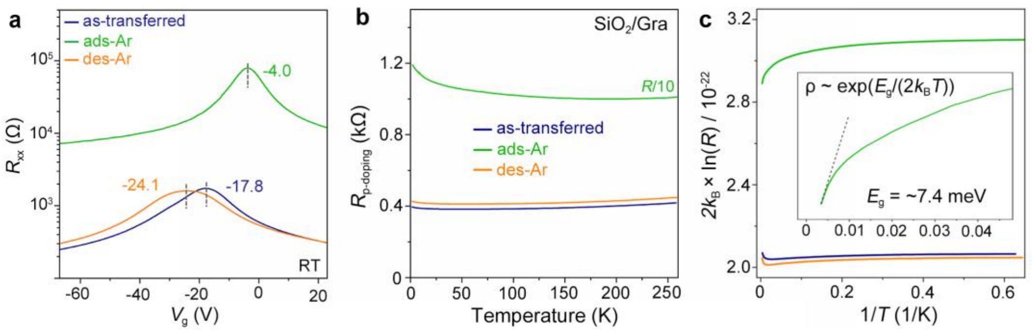

Figure S9. Additional data for variable-temperature resistance of ads-Ar graphene. **a**, The electrical transport of as-transferred, ads-Ar, and des-Ar graphene films on Si\SiO$_2$. **b**, Variable-temperature resistances of as-transferred, ads-Ar and des-Ar graphene films on Si\SiO$_2$, the resistances are collected at the high doping density. The resistances of ads-Ar graphene are divided by tenfold for better comparison. The doping level of graphene is tuned by the gate voltage, the doping density of graphene near CNP is the lowest, and the high doping density is far away from CNP. Here, we use *p*-type doping of +30 V from CNP. **c**, Calculated activation energy of as-transferred, ads-Ar and des-Ar graphene on Si\SiO$_2$, the resistances are collected at CNP and converted from the Fig. S9a. Inset is the fitting for ads-Ar graphene, and the band gap is estimated to be 7.4 meV.

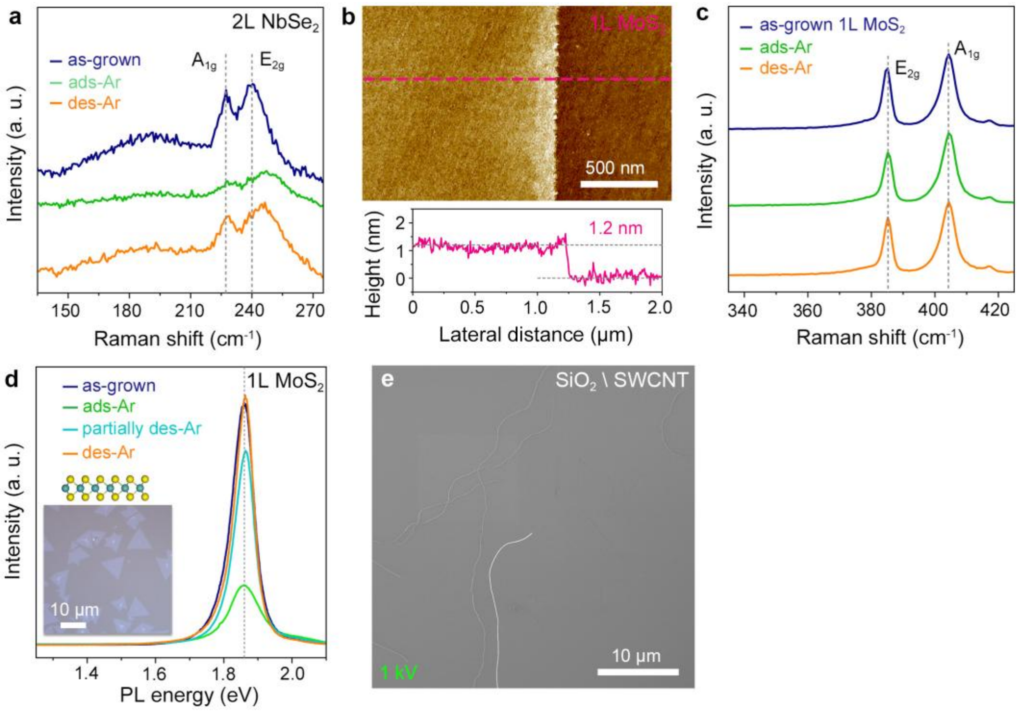

Figure S10. Additional data for stable adsorption of Ar atoms on more low-dimensional materials. **a**, Typical Raman spectra of as-grown, ads-Ar and des-Ar 2L $NbSe_2$ on sapphire. The characteristic peaks of ads-Ar $NbSe_2$ are blueshift and the intensities are reduced. The characteristic peaks of des-Ar $NbSe_2$ can be completely recovered. **b**, Typical AFM image of 1L MoS_2 film on sapphire. They are homogenous with the thickness of 1.2 nm. **c**, Typical Raman spectra of as-grown, ads-Ar and des-Ar 1L MoS_2. The characteristic peaks of as-grown, ads-Ar and des-Ar MoS_2 show no obviously different. **d**, PL spectra of as-grown, ads-Ar, partially des-Ar and des-Ar 1L MoS_2. Inset is the optical image of triangular 1L MoS_2 grains on sapphire. **e**, Typical SEM image of individual SWCNT on Si\SiO_2, the average distance between different SWCNT is <10 μm.
